# Bis(nitrato-κ^2^ *O*,*O*′)bis­[*N*-(triphenyl­meth­yl)pyridin-2-amine-κ*N* ^1^]nickel(II)

**DOI:** 10.1107/S1600536810044624

**Published:** 2010-11-06

**Authors:** Guang-Ning Zhang, Chunjie Jiang

**Affiliations:** a Institute of Chemistry for Functionalized Materials, College of Chemistry and Chemical Engineering, Liaoning Normal University, Dalian 116029, People’s Republic of China

## Abstract

In the title compound, [Ni(NO_3_)_2_(C_24_H_20_N_2_)_2_], the Ni^II^ atom has a distorted pseudo-octa­hedral coordination geometry defined by two chelating nitrate groups and two pyridine N atoms of the monodentate *N*-(triphenyl­meth­yl)pyridin-2-amine ligands. Intra­molecular N—H⋯O hydrogen bonds help to establish the configuration.

## Related literature

For the isostructural dichlorido-cobalt(II), -zinc(II) and -cadmium(II) complexes with bis­{2-[(triphenyl­meth­yl)amino]­pyrid­yl} ligands, see: Fang *et al.* (2006 ▶); Zhang *et al.* (2007 ▶) and Zhang (2008 ▶), respectively. 
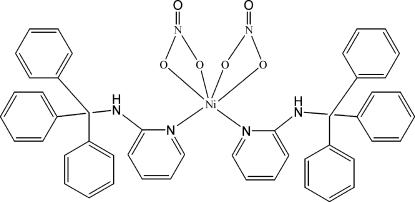


## Experimental

### 

#### Crystal data

[Ni(NO_3_)_2_(C_24_H_20_N_2_)_2_]
*M* *_r_* = 855.57
Monoclinic, 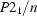

*a* = 10.287 (2) Å
*b* = 23.462 (5) Å
*c* = 17.868 (4) Å
β = 105.266 (3)°
*V* = 4160.3 (14) Å^3^
*Z* = 4
Mo *K*α radiation
μ = 0.53 mm^−1^
*T* = 293 K
0.53 × 0.52 × 0.47 mm

#### Data collection

Bruker SMART APEXII CCD diffractometer
Absorption correction: multi-scan (*SADABS*; Sheldrick, 2003 ▶) *T*_min_ = 0.733, *T*_max_ = 0.791
20535 measured reflections
7279 independent reflections
4928 reflections with *I* > 2σ(*I*)
*R*_int_ = 0.041

#### Refinement

*R*[*F* ^2^ > 2σ(*F* ^2^)] = 0.044
*wR*(*F* ^2^) = 0.114
*S* = 1.01
7279 reflections
550 parameters
H-atom parameters constrained
Δρ_max_ = 0.29 e Å^−3^
Δρ_min_ = −0.30 e Å^−3^

### 

Data collection: *APEX2* (Bruker, 2005 ▶); cell refinement: *SAINT* (Bruker, 2005 ▶); data reduction: *SAINT*; program(s) used to solve structure: *SHELXS97* (Sheldrick, 2008 ▶); program(s) used to refine structure: *SHELXL97* (Sheldrick, 2008 ▶); molecular graphics: *SHELXTL* (Sheldrick, 2008 ▶); software used to prepare material for publication: *SHELXL97*.

## Supplementary Material

Crystal structure: contains datablocks global, I. DOI: 10.1107/S1600536810044624/si2301sup1.cif

Structure factors: contains datablocks I. DOI: 10.1107/S1600536810044624/si2301Isup2.hkl

Additional supplementary materials:  crystallographic information; 3D view; checkCIF report

## Figures and Tables

**Table 1:** Selected bond lengths (Å)

Ni1—O5	2.050 (2)
Ni1—O2	2.0607 (19)
Ni1—N3	2.061 (2)
Ni1—N4	2.062 (2)
Ni1—O4	2.133 (2)
Ni1—O1	2.150 (2)

**Table 2:** Hydrogen-bond geometry (Å, °)

*D*—H⋯*A*	*D*—H	H⋯*A*	*D*⋯*A*	*D*—H⋯*A*
N5—H5*B*⋯O2	0.86	2.16	2.994 (3)	165
N6—H6*A*⋯O5	0.86	2.22	3.048 (3)	162

**Fig. 1.**
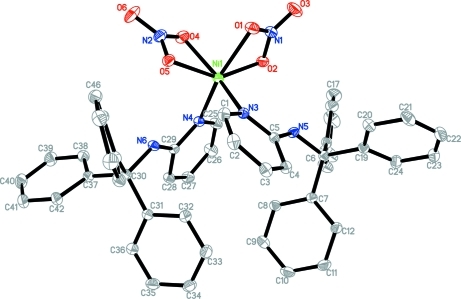
Molecular structure of (I), showing displacement ellipsids at the 30% probability level. H atoms have been omitted.

## supplementary crystallographic information

### Comment

The first example of a distorted tetrahedral metalorganic title related complex, dichloridobis{2-[(triphenylmethyl)amino]pyridyl}cobalt(II), was reported by Fang *et al.* (2006). We continued the study by replacement of cobalt(II) with zinc(II) (Zhang *et al.*, 2007) and cadmium(II) (Zhang, 2008) as the coordination centre. For the title complex we used two nitrato instead of chlorido ligands in order to study the geometric and packing differences for this structure type.

The molecular structure of (I) is shown in Fig.1. In the title compound, the Ni1 atom is distorted pseudo-tetrahedral coordinated by pyridyl atoms N3, N4 and two pairs of nitrato oxygen atoms O1/O2 and O4/O5 (Table 1). The O1—Ni1—O5 and O2—Ni1—O4 angles are observed: 94.26 (9)° and 91.56 (9)°. These two angles are less than the Cl—Co—Cl, Cl—Zn—Cl and Cl—Cd—Cl angles in the literature (121.18 (3)° for Cl-Cd–Cl). The large volume of the two 2-[*N*-(triphenylmethyl)imino]pyridyl ligands determine the crystal packing of the Ni(II) title complex and related dichlorido-Co(II) (Fang *et al.*, 2006), dichlorido-Zn(II) (Zhang *et al.*, 2007) and dichlorido-Cd(II) (Zhang, 2008) structures. The four compounds crystallize in the monoclinic space group *P*2_1_/n and exhibit similar unit-cell parameters. Packing motifs of unit cell projections down the *a* axis are shown in Zhang *et al.* (2007) and Zhang (2008) for this structure type. Intramolecular N—H···O hydrogen bonds help to establish the conformation of the title complex (Table 2). Similar properties are observed with N—H···Cl hydrogen bonds in the dichlorido complexes.

### Experimental

2-[*N*-(triphenylmethyl)imino]pyridyl ligand (0.03 g, 0.09 mmol) and Ni(NO_3_) _2_(0.025 g, 0.14 mmol) were dissolved in 5 ml and 10 ml of ethanol respectively, then mixed. The mixed solution was stirred about 30 minutes and covered with hexane (10 ml). After two months, blue crystals were obtained.

### Refinement

H atoms were positioned geometrically, with N—H=0.86 Å(for NH) and C—H = 0.93 Å for aromatic H, and constrained to ride on their parent atoms, with *U*_iso_(H) = 1.2*U*_eq_(C, N).

### Crystal data

**Table:**

[Ni(NO_3_)_2_(C_24_H_20_N_2_)_2_]	*F*(000) = 1784
*M**_r_* = 855.57	*D*_x_ = 1.366 Mg m^−^^3^
Monoclinic, *P*2_1_/*n*	Mo *K*α radiation, λ = 0.71073 Å
*a* = 10.287 (2) Å	Cell parameters from 3946 reflections
*b* = 23.462 (5) Å	θ = 2.2–21.3°
*c* = 17.868 (4) Å	µ = 0.53 mm^−^^1^
β = 105.266 (3)°	*T* = 293 K
*V* = 4160.3 (14) Å^3^	Plate, blue
*Z* = 4	0.53 × 0.52 × 0.47 mm

### Data collection

**Table:**

Bruker SMART APEXII CCD diffractometer	7279 independent reflections
Radiation source: fine-focus sealed tube	4928 reflections with *I* > 2σ(*I*)
graphite	*R*_int_ = 0.041
CCD scans	θ_max_ = 25.0°, θ_min_ = 2.1°
Absorption correction: multi-scan (*SADABS*; Sheldrick, 2003)	*h* = −11→12
*T*_min_ = 0.733, *T*_max_ = 0.791	*k* = −27→27
20535 measured reflections	*l* = −19→21

### Refinement

**Table:**

Refinement on *F*^2^	Primary atom site location: structure-invariant direct methods
Least-squares matrix: full	Secondary atom site location: difference Fourier map
*R*[*F*^2^ > 2σ(*F*^2^)] = 0.044	Hydrogen site location: inferred from neighbouring sites
*wR*(*F*^2^) = 0.114	H-atom parameters constrained
*S* = 1.01	*w* = 1/[σ^2^(*F*_o_^2^) + (0.0524*P*)^2^ + 0.5431*P*] where *P* = (*F*_o_^2^ + 2*F*_c_^2^)/3
7279 reflections	(Δ/σ)_max_ = 0.001
550 parameters	Δρ_max_ = 0.29 e Å^−^^3^
0 restraints	Δρ_min_ = −0.30 e Å^−^^3^

### Special details

**Table:**

Geometry. All e.s.d.'s (except the e.s.d. in the dihedral angle between two l.s. planes) are estimated using the full covariance matrix. The cell e.s.d.'s are taken into account individually in the estimation of e.s.d.'s in distances, angles and torsion angles; correlations between e.s.d.'s in cell parameters are only used when they are defined by crystal symmetry. An approximate (isotropic) treatment of cell e.s.d.'s is used for estimating e.s.d.'s involving l.s. planes.
Refinement. Refinement of *F*^2^ against ALL reflections. The weighted *R*-factor *wR* and goodness of fit *S* are based on *F*^2^, conventional *R*-factors *R* are based on *F*, with *F* set to zero for negative *F*^2^. The threshold expression of *F*^2^ > σ(*F*^2^) is used only for calculating *R*-factors(gt) *etc*. and is not relevant to the choice of reflections for refinement. *R*-factors based on *F*^2^ are statistically about twice as large as those based on *F*, and *R*- factors based on ALL data will be even larger.

### Fractional atomic coordinates and isotropic or equivalent isotropic displacement parameters (Å^2^)

**Table:**

	*x*	*y*	*z*	*U*_iso_*/*U*_eq_	
Ni1	0.68497 (3)	0.181447 (15)	0.17328 (2)	0.04318 (13)	
O1	0.7259 (2)	0.21664 (10)	0.07075 (13)	0.0656 (6)	
O2	0.83125 (19)	0.24336 (8)	0.18590 (12)	0.0556 (5)	
O3	0.8942 (3)	0.27540 (12)	0.08652 (16)	0.0931 (9)	
O4	0.8238 (2)	0.11640 (10)	0.16253 (14)	0.0660 (6)	
O5	0.6099 (2)	0.10632 (8)	0.12069 (11)	0.0545 (5)	
O6	0.7416 (3)	0.03988 (11)	0.09769 (16)	0.0996 (9)	
C1	0.4878 (3)	0.27695 (11)	0.18500 (15)	0.0393 (6)	
C2	0.3571 (3)	0.29778 (12)	0.17711 (17)	0.0475 (7)	
H2A	0.3441	0.3316	0.2012	0.057*	
C3	0.2494 (3)	0.26823 (13)	0.13411 (18)	0.0530 (8)	
H3A	0.1626	0.2819	0.1289	0.064*	
C4	0.2688 (3)	0.21793 (13)	0.09802 (18)	0.0571 (8)	
H4A	0.1964	0.1979	0.0669	0.068*	
C5	0.3968 (3)	0.19888 (13)	0.10973 (17)	0.0526 (8)	
H5A	0.4102	0.1649	0.0860	0.063*	
C6	0.6152 (3)	0.13579 (11)	0.32002 (16)	0.0404 (6)	
C7	0.6306 (3)	0.13566 (13)	0.39981 (16)	0.0492 (7)	
H7A	0.5686	0.1167	0.4204	0.059*	
C8	0.7370 (3)	0.16348 (14)	0.44772 (18)	0.0570 (8)	
H8A	0.7476	0.1634	0.5010	0.068*	
C9	0.8285 (3)	0.19170 (14)	0.41694 (19)	0.0636 (9)	
H9A	0.9024	0.2103	0.4488	0.076*	
C10	0.8075 (3)	0.19145 (13)	0.33879 (17)	0.0541 (8)	
H10A	0.8692	0.2106	0.3181	0.065*	
C11	0.6099 (3)	0.35901 (11)	0.26601 (16)	0.0413 (7)	
C12	0.3994 (3)	0.07672 (11)	0.28363 (15)	0.0407 (7)	
C13	0.5717 (3)	0.41085 (12)	0.21192 (17)	0.0453 (7)	
C14	0.5979 (3)	0.46533 (13)	0.2424 (2)	0.0597 (8)	
H14A	0.6378	0.4699	0.2952	0.072*	
C15	0.5654 (4)	0.51291 (15)	0.1954 (2)	0.0732 (10)	
H15A	0.5829	0.5492	0.2167	0.088*	
C16	0.5082 (4)	0.50679 (18)	0.1184 (3)	0.0794 (11)	
H16A	0.4854	0.5389	0.0871	0.095*	
C17	0.4839 (4)	0.45370 (18)	0.0866 (2)	0.0762 (11)	
H17A	0.4452	0.4497	0.0337	0.091*	
C18	0.5166 (3)	0.40572 (14)	0.13285 (19)	0.0586 (8)	
H18A	0.5013	0.3697	0.1105	0.070*	
C19	0.5259 (3)	0.35298 (12)	0.32558 (16)	0.0413 (7)	
N1	0.8190 (3)	0.24611 (12)	0.11230 (18)	0.0611 (7)	
N2	0.7279 (3)	0.08570 (13)	0.12585 (16)	0.0633 (7)	
N3	0.5067 (2)	0.22612 (9)	0.15372 (12)	0.0408 (5)	
N4	0.7028 (2)	0.16517 (9)	0.28896 (13)	0.0411 (5)	
N5	0.6013 (2)	0.30583 (9)	0.22205 (13)	0.0428 (6)	
H5B	0.6765	0.2912	0.2193	0.051*	
N6	0.5169 (2)	0.10664 (9)	0.26913 (13)	0.0425 (6)	
H6A	0.5236	0.1054	0.2222	0.051*	
C20	0.4470 (3)	0.39598 (13)	0.34225 (18)	0.0525 (8)	
H20A	0.4366	0.4298	0.3142	0.063*	
C21	0.3834 (3)	0.38893 (16)	0.4007 (2)	0.0714 (10)	
H21A	0.3313	0.4184	0.4121	0.086*	
C22	0.3957 (3)	0.33925 (16)	0.4420 (2)	0.0686 (10)	
H22A	0.3525	0.3350	0.4813	0.082*	
C23	0.4724 (3)	0.29587 (15)	0.42496 (18)	0.0589 (8)	
H23A	0.4811	0.2619	0.4526	0.071*	
C24	0.5366 (3)	0.30271 (12)	0.36678 (17)	0.0484 (7)	
H24A	0.5879	0.2730	0.3552	0.058*	
C25	0.7599 (3)	0.36597 (12)	0.31007 (17)	0.0451 (7)	
C26	0.8008 (3)	0.37622 (16)	0.3884 (2)	0.0721 (10)	
H26A	0.7371	0.3781	0.4168	0.087*	
C27	0.9352 (4)	0.3837 (2)	0.4253 (2)	0.1057 (16)	
H27A	0.9614	0.3917	0.4781	0.127*	
C28	1.0291 (4)	0.3796 (2)	0.3850 (3)	0.1009 (15)	
H28A	1.1200	0.3828	0.4106	0.121*	
C29	0.9910 (4)	0.37064 (17)	0.3065 (3)	0.0862 (12)	
H29A	1.0556	0.3685	0.2787	0.103*	
C30	0.8563 (3)	0.36475 (13)	0.2689 (2)	0.0624 (9)	
H30A	0.8301	0.3599	0.2153	0.075*	
C31	0.3252 (3)	0.11809 (12)	0.32560 (16)	0.0422 (7)	
C32	0.2881 (3)	0.10467 (13)	0.39223 (18)	0.0539 (8)	
H32A	0.3092	0.0690	0.4149	0.065*	
C33	0.2197 (3)	0.14399 (15)	0.4256 (2)	0.0660 (9)	
H33A	0.1953	0.1344	0.4706	0.079*	
C34	0.1878 (3)	0.19659 (15)	0.3933 (2)	0.0644 (9)	
H34A	0.1416	0.2227	0.4160	0.077*	
C35	0.2242 (3)	0.21059 (14)	0.3277 (2)	0.0647 (9)	
H35A	0.2023	0.2462	0.3051	0.078*	
C36	0.2935 (3)	0.17186 (13)	0.29476 (19)	0.0554 (8)	
H36A	0.3196	0.1822	0.2506	0.066*	
C37	0.3108 (3)	0.06214 (11)	0.20148 (16)	0.0426 (7)	
C38	0.1758 (3)	0.07570 (13)	0.17557 (18)	0.0530 (8)	
H38A	0.1325	0.0930	0.2091	0.064*	
C39	0.1050 (3)	0.06387 (15)	0.1007 (2)	0.0650 (9)	
H39A	0.0138	0.0728	0.0842	0.078*	
C40	0.1669 (4)	0.03915 (14)	0.0502 (2)	0.0655 (9)	
H40A	0.1193	0.0328	−0.0011	0.079*	
C41	0.2990 (3)	0.02375 (13)	0.07534 (19)	0.0612 (9)	
H41A	0.3414	0.0064	0.0413	0.073*	
C42	0.3699 (3)	0.03371 (12)	0.15082 (17)	0.0519 (8)	
H42A	0.4586	0.0212	0.1682	0.062*	
C43	0.4392 (3)	0.02037 (11)	0.32687 (16)	0.0423 (7)	
C44	0.3388 (3)	−0.01635 (13)	0.33673 (17)	0.0530 (8)	
H44A	0.2488	−0.0065	0.3165	0.064*	
C45	0.3707 (4)	−0.06708 (14)	0.3760 (2)	0.0665 (9)	
H45A	0.3021	−0.0906	0.3829	0.080*	
C46	0.5015 (4)	−0.08334 (15)	0.4049 (2)	0.0716 (10)	
H46A	0.5225	−0.1173	0.4321	0.086*	
C47	0.6017 (4)	−0.04883 (15)	0.3930 (2)	0.0705 (10)	
H47A	0.6912	−0.0599	0.4114	0.085*	
C48	0.5707 (3)	0.00223 (13)	0.35413 (18)	0.0562 (8)	
H48A	0.6399	0.0249	0.3461	0.067*	

### Atomic displacement parameters (Å^2^)

**Table:**

	*U*^11^	*U*^22^	*U*^33^	*U*^12^	*U*^13^	*U*^23^
Ni1	0.0444 (2)	0.0463 (2)	0.0411 (2)	−0.00194 (17)	0.01515 (17)	−0.00311 (17)
O1	0.0695 (15)	0.0810 (16)	0.0498 (14)	−0.0109 (13)	0.0223 (12)	−0.0027 (12)
O2	0.0553 (13)	0.0595 (13)	0.0568 (15)	−0.0055 (10)	0.0233 (11)	−0.0067 (11)
O3	0.098 (2)	0.099 (2)	0.099 (2)	−0.0251 (16)	0.0539 (17)	0.0166 (16)
O4	0.0573 (14)	0.0686 (15)	0.0732 (16)	0.0058 (12)	0.0190 (12)	−0.0129 (13)
O5	0.0578 (14)	0.0532 (13)	0.0527 (13)	−0.0010 (11)	0.0152 (11)	−0.0071 (10)
O6	0.128 (2)	0.0669 (17)	0.102 (2)	0.0222 (16)	0.0274 (17)	−0.0336 (16)
C1	0.0405 (16)	0.0413 (16)	0.0351 (16)	−0.0014 (13)	0.0085 (13)	0.0023 (13)
C2	0.0410 (18)	0.0467 (17)	0.0522 (19)	0.0032 (14)	0.0077 (14)	0.0053 (14)
C3	0.0402 (18)	0.059 (2)	0.054 (2)	0.0035 (15)	0.0021 (14)	0.0142 (16)
C4	0.049 (2)	0.056 (2)	0.054 (2)	−0.0098 (16)	−0.0073 (15)	0.0042 (16)
C5	0.054 (2)	0.0521 (19)	0.0468 (19)	−0.0041 (15)	0.0043 (15)	−0.0056 (15)
C6	0.0386 (16)	0.0419 (16)	0.0406 (17)	0.0023 (13)	0.0104 (13)	−0.0033 (13)
C7	0.0486 (18)	0.0594 (19)	0.0399 (18)	−0.0063 (15)	0.0124 (14)	−0.0010 (15)
C8	0.057 (2)	0.073 (2)	0.0378 (18)	−0.0028 (17)	0.0073 (15)	−0.0057 (16)
C9	0.051 (2)	0.086 (3)	0.047 (2)	−0.0190 (17)	0.0004 (15)	−0.0067 (17)
C10	0.0410 (17)	0.071 (2)	0.049 (2)	−0.0131 (15)	0.0099 (15)	−0.0019 (16)
C11	0.0383 (16)	0.0398 (16)	0.0454 (17)	−0.0019 (12)	0.0101 (13)	−0.0033 (13)
C12	0.0421 (16)	0.0418 (16)	0.0397 (16)	−0.0035 (13)	0.0132 (13)	−0.0011 (13)
C13	0.0384 (16)	0.0505 (18)	0.0488 (19)	0.0000 (13)	0.0144 (14)	0.0034 (15)
C14	0.070 (2)	0.048 (2)	0.061 (2)	0.0015 (16)	0.0172 (17)	0.0029 (17)
C15	0.084 (3)	0.050 (2)	0.096 (3)	0.0018 (18)	0.043 (2)	0.010 (2)
C16	0.085 (3)	0.068 (3)	0.095 (3)	0.018 (2)	0.042 (2)	0.034 (2)
C17	0.080 (3)	0.091 (3)	0.057 (2)	0.001 (2)	0.0185 (19)	0.027 (2)
C18	0.061 (2)	0.060 (2)	0.057 (2)	−0.0063 (16)	0.0188 (17)	0.0061 (17)
C19	0.0325 (15)	0.0474 (17)	0.0432 (17)	−0.0051 (13)	0.0087 (12)	−0.0058 (14)
N1	0.0648 (19)	0.0613 (18)	0.066 (2)	−0.0014 (15)	0.0325 (16)	0.0043 (15)
N2	0.078 (2)	0.0576 (19)	0.0553 (18)	0.0114 (17)	0.0187 (16)	−0.0052 (15)
N3	0.0425 (14)	0.0441 (14)	0.0345 (13)	−0.0003 (11)	0.0080 (11)	−0.0007 (11)
N4	0.0339 (13)	0.0483 (14)	0.0403 (14)	−0.0022 (10)	0.0085 (11)	−0.0015 (11)
N5	0.0343 (13)	0.0444 (14)	0.0501 (15)	0.0017 (10)	0.0116 (11)	−0.0081 (11)
N6	0.0426 (13)	0.0522 (14)	0.0340 (13)	−0.0088 (11)	0.0125 (11)	−0.0026 (11)
C20	0.0459 (18)	0.0470 (18)	0.069 (2)	−0.0013 (14)	0.0224 (16)	−0.0036 (15)
C21	0.058 (2)	0.066 (2)	0.103 (3)	−0.0010 (18)	0.044 (2)	−0.016 (2)
C22	0.062 (2)	0.084 (3)	0.072 (2)	−0.017 (2)	0.0383 (19)	−0.011 (2)
C23	0.0515 (19)	0.069 (2)	0.056 (2)	−0.0105 (17)	0.0125 (16)	0.0095 (17)
C24	0.0420 (17)	0.0520 (19)	0.0526 (19)	0.0002 (14)	0.0149 (14)	0.0020 (15)
C25	0.0407 (16)	0.0427 (17)	0.0506 (19)	−0.0059 (13)	0.0098 (14)	0.0013 (14)
C26	0.050 (2)	0.108 (3)	0.053 (2)	−0.0233 (19)	0.0054 (16)	0.004 (2)
C27	0.065 (3)	0.168 (5)	0.070 (3)	−0.044 (3)	−0.007 (2)	0.021 (3)
C28	0.042 (2)	0.139 (4)	0.107 (4)	−0.023 (2)	−0.005 (2)	0.023 (3)
C29	0.048 (2)	0.101 (3)	0.115 (4)	−0.017 (2)	0.030 (2)	−0.011 (3)
C30	0.0460 (19)	0.069 (2)	0.075 (2)	−0.0109 (16)	0.0199 (17)	−0.0081 (18)
C31	0.0402 (16)	0.0474 (17)	0.0379 (16)	−0.0058 (13)	0.0084 (13)	−0.0039 (14)
C32	0.0589 (19)	0.0527 (19)	0.053 (2)	0.0003 (15)	0.0193 (16)	0.0007 (15)
C33	0.070 (2)	0.077 (3)	0.059 (2)	−0.0062 (19)	0.0326 (18)	−0.0098 (19)
C34	0.058 (2)	0.061 (2)	0.080 (3)	−0.0018 (17)	0.0297 (19)	−0.0191 (19)
C35	0.066 (2)	0.050 (2)	0.079 (3)	0.0017 (17)	0.0206 (19)	−0.0037 (18)
C36	0.064 (2)	0.051 (2)	0.055 (2)	−0.0001 (16)	0.0232 (16)	−0.0024 (15)
C37	0.0432 (17)	0.0407 (16)	0.0442 (17)	−0.0090 (13)	0.0122 (13)	0.0029 (13)
C38	0.0500 (19)	0.0558 (19)	0.052 (2)	−0.0069 (15)	0.0112 (15)	−0.0018 (15)
C39	0.048 (2)	0.072 (2)	0.065 (2)	−0.0051 (17)	−0.0020 (17)	−0.0020 (19)
C40	0.073 (3)	0.068 (2)	0.047 (2)	−0.0184 (19)	0.0016 (18)	−0.0012 (17)
C41	0.068 (2)	0.061 (2)	0.057 (2)	−0.0164 (18)	0.0189 (18)	−0.0173 (17)
C42	0.0513 (19)	0.0510 (19)	0.052 (2)	−0.0061 (15)	0.0119 (15)	−0.0088 (15)
C43	0.0446 (18)	0.0426 (16)	0.0407 (16)	−0.0005 (13)	0.0128 (13)	0.0014 (13)
C44	0.0511 (19)	0.0503 (19)	0.057 (2)	−0.0014 (15)	0.0133 (15)	0.0083 (16)
C45	0.081 (3)	0.049 (2)	0.073 (2)	−0.0089 (18)	0.026 (2)	0.0060 (18)
C46	0.088 (3)	0.051 (2)	0.072 (3)	0.010 (2)	0.015 (2)	0.0142 (18)
C47	0.067 (2)	0.060 (2)	0.081 (3)	0.0165 (19)	0.0123 (19)	0.0123 (19)
C48	0.052 (2)	0.055 (2)	0.063 (2)	−0.0017 (16)	0.0175 (16)	−0.0007 (16)

### Geometric parameters (Å, °)

**Table:**

Ni1—O5	2.050 (2)	N5—H5B	0.8600
Ni1—O2	2.0607 (19)	N6—H6A	0.8600
Ni1—N3	2.061 (2)	C20—C21	1.380 (4)
Ni1—N4	2.062 (2)	C20—H20A	0.9300
Ni1—O4	2.133 (2)	C21—C22	1.368 (5)
Ni1—O1	2.150 (2)	C21—H21A	0.9300
O1—N1	1.254 (3)	C22—C23	1.370 (4)
O2—N1	1.289 (3)	C22—H22A	0.9300
O3—N1	1.213 (3)	C23—C24	1.380 (4)
O4—N2	1.258 (3)	C23—H23A	0.9300
O5—N2	1.287 (3)	C24—H24A	0.9300
O6—N2	1.211 (3)	C25—C26	1.372 (4)
C1—N3	1.353 (3)	C25—C30	1.382 (4)
C1—N5	1.361 (3)	C26—C27	1.377 (5)
C1—C2	1.403 (3)	C26—H26A	0.9300
C2—C3	1.360 (4)	C27—C28	1.353 (6)
C2—H2A	0.9300	C27—H27A	0.9300
C3—C4	1.384 (4)	C28—C29	1.369 (6)
C3—H3A	0.9300	C28—H28A	0.9300
C4—C5	1.354 (4)	C29—C30	1.379 (5)
C4—H4A	0.9300	C29—H29A	0.9300
C5—N3	1.355 (3)	C30—H30A	0.9300
C5—H5A	0.9300	C31—C32	1.379 (4)
C6—N6	1.354 (3)	C31—C36	1.381 (4)
C6—N4	1.363 (3)	C32—C33	1.387 (4)
C6—C7	1.392 (4)	C32—H32A	0.9300
C7—C8	1.365 (4)	C33—C34	1.365 (5)
C7—H7A	0.9300	C33—H33A	0.9300
C8—C9	1.379 (4)	C34—C35	1.362 (5)
C8—H8A	0.9300	C34—H34A	0.9300
C9—C10	1.356 (4)	C35—C36	1.379 (4)
C9—H9A	0.9300	C35—H35A	0.9300
C10—N4	1.352 (3)	C36—H36A	0.9300
C10—H10A	0.9300	C37—C38	1.380 (4)
C11—N5	1.464 (3)	C37—C42	1.387 (4)
C11—C13	1.539 (4)	C38—C39	1.373 (4)
C11—C19	1.545 (4)	C38—H38A	0.9300
C11—C25	1.545 (4)	C39—C40	1.363 (5)
C12—N6	1.478 (3)	C39—H39A	0.9300
C12—C43	1.532 (4)	C40—C41	1.363 (4)
C12—C31	1.546 (4)	C40—H40A	0.9300
C12—C37	1.549 (4)	C41—C42	1.374 (4)
C13—C18	1.382 (4)	C41—H41A	0.9300
C13—C14	1.388 (4)	C42—H42A	0.9300
C14—C15	1.384 (4)	C43—C48	1.379 (4)
C14—H14A	0.9300	C43—C44	1.391 (4)
C15—C16	1.354 (5)	C44—C45	1.377 (4)
C15—H15A	0.9300	C44—H44A	0.9300
C16—C17	1.364 (5)	C45—C46	1.362 (5)
C16—H16A	0.9300	C45—H45A	0.9300
C17—C18	1.384 (4)	C46—C47	1.370 (5)
C17—H17A	0.9300	C46—H46A	0.9300
C18—H18A	0.9300	C47—C48	1.380 (4)
C19—C20	1.375 (4)	C47—H47A	0.9300
C19—C24	1.379 (4)	C48—H48A	0.9300
			
O5—Ni1—O2	146.32 (8)	C1—N5—C11	127.5 (2)
O5—Ni1—N3	98.53 (9)	C1—N5—H5B	116.3
O2—Ni1—N3	104.58 (8)	C11—N5—H5B	116.3
O5—Ni1—N4	102.92 (8)	C6—N6—C12	128.5 (2)
O2—Ni1—N4	98.69 (9)	C6—N6—H6A	115.7
N3—Ni1—N4	96.00 (8)	C12—N6—H6A	115.7
O5—Ni1—O4	61.58 (8)	C19—C20—C21	120.0 (3)
O2—Ni1—O4	91.55 (9)	C19—C20—H20A	120.0
N3—Ni1—O4	159.34 (9)	C21—C20—H20A	120.0
N4—Ni1—O4	94.17 (9)	C22—C21—C20	121.0 (3)
O5—Ni1—O1	94.26 (9)	C22—C21—H21A	119.5
O2—Ni1—O1	61.40 (8)	C20—C21—H21A	119.5
N3—Ni1—O1	91.46 (9)	C21—C22—C23	119.3 (3)
N4—Ni1—O1	159.99 (9)	C21—C22—H22A	120.3
O4—Ni1—O1	84.95 (9)	C23—C22—H22A	120.3
N1—O1—Ni1	89.87 (18)	C22—C23—C24	119.9 (3)
N1—O2—Ni1	92.94 (17)	C22—C23—H23A	120.0
N2—O4—Ni1	90.24 (17)	C24—C23—H23A	120.0
N2—O5—Ni1	93.21 (17)	C19—C24—C23	120.9 (3)
N3—C1—N5	116.2 (2)	C19—C24—H24A	119.5
N3—C1—C2	120.3 (2)	C23—C24—H24A	119.5
N5—C1—C2	123.5 (3)	C26—C25—C30	118.5 (3)
C3—C2—C1	119.7 (3)	C26—C25—C11	122.4 (3)
C3—C2—H2A	120.1	C30—C25—C11	119.1 (3)
C1—C2—H2A	120.1	C25—C26—C27	120.7 (3)
C2—C3—C4	120.2 (3)	C25—C26—H26A	119.7
C2—C3—H3A	119.9	C27—C26—H26A	119.7
C4—C3—H3A	119.9	C28—C27—C26	120.3 (4)
C5—C4—C3	117.6 (3)	C28—C27—H27A	119.9
C5—C4—H4A	121.2	C26—C27—H27A	119.9
C3—C4—H4A	121.2	C27—C28—C29	120.3 (4)
C4—C5—N3	124.3 (3)	C27—C28—H28A	119.9
C4—C5—H5A	117.9	C29—C28—H28A	119.9
N3—C5—H5A	117.9	C28—C29—C30	119.7 (4)
N6—C6—N4	116.0 (2)	C28—C29—H29A	120.2
N6—C6—C7	123.4 (2)	C30—C29—H29A	120.2
N4—C6—C7	120.6 (3)	C29—C30—C25	120.5 (3)
C8—C7—C6	119.8 (3)	C29—C30—H30A	119.7
C8—C7—H7A	120.1	C25—C30—H30A	119.7
C6—C7—H7A	120.1	C32—C31—C36	117.6 (3)
C7—C8—C9	119.9 (3)	C32—C31—C12	124.0 (3)
C7—C8—H8A	120.1	C36—C31—C12	118.3 (2)
C9—C8—H8A	120.1	C31—C32—C33	120.4 (3)
C10—C9—C8	117.9 (3)	C31—C32—H32A	119.8
C10—C9—H9A	121.0	C33—C32—H32A	119.8
C8—C9—H9A	121.0	C34—C33—C32	120.8 (3)
N4—C10—C9	124.3 (3)	C34—C33—H33A	119.6
N4—C10—H10A	117.8	C32—C33—H33A	119.6
C9—C10—H10A	117.8	C35—C34—C33	119.5 (3)
N5—C11—C13	111.5 (2)	C35—C34—H34A	120.3
N5—C11—C19	109.0 (2)	C33—C34—H34A	120.3
C13—C11—C19	114.1 (2)	C34—C35—C36	119.9 (3)
N5—C11—C25	106.0 (2)	C34—C35—H35A	120.0
C13—C11—C25	107.0 (2)	C36—C35—H35A	120.0
C19—C11—C25	108.9 (2)	C35—C36—C31	121.7 (3)
N6—C12—C43	112.1 (2)	C35—C36—H36A	119.1
N6—C12—C31	108.5 (2)	C31—C36—H36A	119.1
C43—C12—C31	113.4 (2)	C38—C37—C42	117.9 (3)
N6—C12—C37	104.1 (2)	C38—C37—C12	123.8 (3)
C43—C12—C37	107.5 (2)	C42—C37—C12	118.3 (3)
C31—C12—C37	110.8 (2)	C39—C38—C37	120.6 (3)
C18—C13—C14	117.8 (3)	C39—C38—H38A	119.7
C18—C13—C11	122.8 (3)	C37—C38—H38A	119.7
C14—C13—C11	119.4 (3)	C40—C39—C38	120.7 (3)
C15—C14—C13	120.9 (3)	C40—C39—H39A	119.7
C15—C14—H14A	119.6	C38—C39—H39A	119.7
C13—C14—H14A	119.6	C41—C40—C39	119.6 (3)
C16—C15—C14	120.2 (4)	C41—C40—H40A	120.2
C16—C15—H15A	119.9	C39—C40—H40A	120.2
C14—C15—H15A	119.9	C40—C41—C42	120.2 (3)
C15—C16—C17	120.2 (4)	C40—C41—H41A	119.9
C15—C16—H16A	119.9	C42—C41—H41A	119.9
C17—C16—H16A	119.9	C41—C42—C37	120.8 (3)
C16—C17—C18	120.4 (4)	C41—C42—H42A	119.6
C16—C17—H17A	119.8	C37—C42—H42A	119.6
C18—C17—H17A	119.8	C48—C43—C44	117.1 (3)
C13—C18—C17	120.6 (3)	C48—C43—C12	123.5 (2)
C13—C18—H18A	119.7	C44—C43—C12	119.3 (2)
C17—C18—H18A	119.7	C45—C44—C43	120.9 (3)
C20—C19—C24	118.7 (3)	C45—C44—H44A	119.5
C20—C19—C11	123.5 (3)	C43—C44—H44A	119.5
C24—C19—C11	117.7 (2)	C46—C45—C44	121.0 (3)
O3—N1—O1	123.5 (3)	C46—C45—H45A	119.5
O3—N1—O2	121.0 (3)	C44—C45—H45A	119.5
O1—N1—O2	115.5 (2)	C45—C46—C47	118.9 (3)
O6—N2—O4	124.3 (3)	C45—C46—H46A	120.5
O6—N2—O5	121.0 (3)	C47—C46—H46A	120.5
O4—N2—O5	114.7 (3)	C46—C47—C48	120.5 (3)
C1—N3—C5	117.8 (2)	C46—C47—H47A	119.8
C1—N3—Ni1	126.62 (18)	C48—C47—H47A	119.8
C5—N3—Ni1	115.33 (19)	C43—C48—C47	121.4 (3)
C10—N4—C6	117.4 (2)	C43—C48—H48A	119.3
C10—N4—Ni1	114.90 (18)	C47—C48—H48A	119.3
C6—N4—Ni1	127.43 (18)		

### Hydrogen-bond geometry (Å, °)

**Table:**

*D*—H···*A*	*D*—H	H···*A*	*D*···*A*	*D*—H···*A*
N5—H5B···O2	0.86	2.16	2.994 (3)	165
N6—H6A···O5	0.86	2.22	3.048 (3)	162
